# Evaluation of Immune Exhaustion and Co-Inhibitory Receptor Expression in *Mycobacterium avium* Subspecies *paratuberculosis* (MAP) Seropositive Diarrhoeic Bovines

**DOI:** 10.3390/pathogens13060473

**Published:** 2024-06-04

**Authors:** Shalini Sharma, Khushbu Sharma, Ram Kumar, Deen Dayal, Shweta Dhanda, Naveen Kumar, Kundan Kumar Chaubey, Shoor Vir Singh, Sikander Banger, Vishal Sharma

**Affiliations:** 1 Department of Veterinary Physiology and Biochemistry, Lala Lajpat Rai University of Veterinary and Animal Sciences, Hisar 125004, India; khushbu181997@gmail.com; 2 National Centre for Veterinary Type Cultures, ICAR-NRC on Equines Sirsa Road, Hisar 125001, India; ramkumarjangir01@gmail.com (R.K.); shwetad2292@gmail.com (S.D.); naveenkumar.icar@gmail.com (N.K.); 3 Department of Bio-Technology, GLA University, Post-Chaumuhan, Mathura 281406, India; dayald082@gmail.com (D.D.); shoorvir.singh@gla.ac.in (S.V.S.); 4 School of Basic and Applied Sciences, Sanskriti University, Mathura 281401, India; kundan2006chaubey@gmail.com; 5 Department of Veterinary Medicine, Lala Lajpat Rai University of Veterinary and Animal Sciences, Hisar 125004, India; sikander2019luvas@gmail.com; 6 Department of Livestock Production Management, Lala Lajpat Rai University of Veterinary and Animal Sciences, Hisar 125004, India; drvishalsharma@luvas.edu.in

**Keywords:** immune-exhaustion, TIM-3, PD-1, *Mycobacterium avium* subspecies *paratuberculosis*, immunoinhibitory receptors, buffaloes, stray cattle

## Abstract

*Mycobacterium avium* subspecies *paratuberculosis* (MAP) infection leads to chronic, persistent granulomatous enteritis, causing prolonged diarrhoea and emaciation. The disease is managed using medications such as antibiotics, live vaccines, mycobacteriophage therapies and other treatments; however, a notable proportion of affected animals do not show improvement with this approach. We hypothesise that immunoinhibitory receptors TIM-3 (T cell immunoglobulin mucin protein-3) and PD-1 (Programmed death receptor 1) may be upregulated on Peripheral blood mononuclear cells (PBMCs) of MAP-seropositive bovines, potentially contributing to immune exhaustion. Samples (blood and faeces) were collected from 32 diarrhoeic bovines suspected of MAP infection; eight apparently healthy buffaloes from the dairy farm at Hisar, Haryana and from 14 cows (suffering from chronic diarrhoea, weakness and emaciation) housed in stray cattle shed. MAP infection was estimated using indigenous ELISA (i-ELISA), faecal IS*900* PCR, culture and acid-fast staining. TIM-3 and PD-1 gene expression on PBMCs were determined using qRT-PCR. TIM3 expression was relatively higher (~400-fold, 330-fold, 112-fold, 65-fold and 16-fold) in 5 chronically diarrhoeic PBMCs samples (MAP-seropositive), and higher PD-1 expression (around ~7-fold, 1.75-fold, 2.5-fold, 7.6-fold) was recorded in 4 diarrhoeic MAP-seropositive animals, compared to apparently healthy and other MAP-seronegative diarrhoeic animals. High co-expression of TIM-3 and PD-1 levels was also recorded in chronically diarrhoeic, emaciated stray cattle. Understanding immune responses in field conditions might aid in the therapeutic management of *paratuberculosis.*

## 1. Introduction

Infection of MAP in buffaloes leads to high morbidity (weakness and diarrhoea) and enormous losses in productivity [1]. Since MAP bacilli survive pasteurisation, therefore milk is a potential source of human infection. Consumption of dairy products prepared from pasteurised milk poses serious public health hazards [2]. Animals develop chronic granulomatous enteritis, resulting in long-standing diarrhoea [3]. A significant number of buffaloes suffering from chronic diarrhoea do not respond to the anti-diarrhoeal drugs, which progresses to loss in body condition and emaciation. Management of MAP infection typically involves a multifaceted approach, including antibiotic treatment, live vaccines, and mycobacteriophage therapies aimed at controlling the disease in affected animals [4]. However, definitive treatment for MAP infection in humans remains elusive. Recent advancements in diagnostics, such as PCR and serological assays, have enhanced the detection of MAP [5]. Furthermore, emerging therapies targeting MAP, including novel antibiotics and immunomodulatory agents, hold promise in addressing the challenges posed by this chronic infection [6]. These developments highlight the importance of further research to understand the intricate relationship between immune regulation, infectious agents like MAP, and potential therapeutic interventions [7].

‘T cell exhaustion’ has been shown to occur in chronic viral infection [8], but more recently, in the case of immune response to tumours [9,10,11], autoimmunity [12,13], and also bacterial infections [14,15]. Characterising the molecular mechanisms and pathophysiology of exhaustion may have substantial implications in the successful blockade of immunoinhibitory interactions and adoptive T cell transfer therapeutics. T cell exhaustion leads to diminished cytokines production upon TCR (T cell receptor) activation, reduced proliferation, enhanced immunoinhibitory receptor expression, and exhausted cells become more prone to apoptosis. T cell exhaustion studies in cattle have elucidated the roles of immunoinhibitory molecules such as TIM-3 and PD-1/programmed death-ligand-1 (PD-L1) in immune exhaustion and disease progression in bovine leukaemia virus (BLV) infection, *paratuberculosis* and anaplasmosis [16]. PD-L1 expressing cells increase following infection, and upon PD-L1 blockade, antiviral immune responses were restored in-vitro [16]. MAP-specific T Cell Responses [14] are also known to be inhibited by bovine immunoinhibitory receptors. Both mRNA and protein expression of PD-1 and PD-L1 were stimulated in case of cytopathic (CP) BVDV (bovine viral diarrhoea virus) (strain NADL) and non-cytopathic (NCP) BVDV (strain KD) infection. TIM-3, an immune checkpoint receptor, contributes to immune exhaustion in chronic infections and cancer by suppressing immune responses through interactions with its ligands [17]. Upregulated on exhausted T cells, TIM-3 leads to reduced effector function, cytokine production, and proliferation. Targeting TIM-3 signalling is a potential strategy to reverse immune exhaustion and enhance antitumor or antiviral immunity. The up-regulation of TIM-3 and PD-1/PD-L1 was accompanied by decreased peripheral blood lymphocyte (PBL) proliferation and enhanced apoptosis [17].

However, the majority of these studies have been done in experimentally infected cattle models. Here, we are reporting immune exhaustion in spontaneous cases of diarrhoeic buffaloes to veterinary clinics to seek therapeutic intervention. These buffaloes were suspected of MAP infection; however, under field conditions, usually more than one etiological agent may be involved in cases of diarrhoea. Understanding immune responses in such cases will highlight the general progression of the disease and the immunological status of the animal. This may have both diagnostic and prognostic value on one hand, while on the other, it might aid in developing a new therapeutic regime or will help to increase the efficiency of the present regime with judicial uses. A balanced immune response to various pathogens, apt chemokine and cytokines milieu, and the activation of regulatory T cells are vital for the survival of the vertebrate host in natural settings where co-infections are quite common. In these complex clinical scenarios, where multiple etiological agents may be involved, it would be worth estimating the status of host immune responses in spontaneous cases of diarrhoeic buffaloes suspected of MAP infection. Elucidating such mechanisms will lead to a paradigm shift in the development of anti-diarrhoeal treatment regimes in terms of improving immune responses to counter-balance pathological changes. We, therefore, aimed to evaluate the co-expression of immunoinhibitory receptors TIM-3 and PD-1 on PBMCs fraction of diarrhoeic bovines and whether the high or low expression levels correlate with MAP infection.

## 2. Materials and Methods

### 2.1. Sample Collection

Diarrhoeic animals (buffaloes and cows) reporting at the Veterinary Clinical Complex (VCC) of Lala Lajpat Rai University of Veterinary and Animal Sciences (LUVAS), Hisar, Haryana, were included in the study. Blood and faecal samples were collected from 32 diarrhoeic buffaloes suspected of MAP infection. Animals reported for treatment at veterinary clinics had clinical presentations of chronic diarrhoea. The diarrhoeic condition had a variable presentation (hard to lose faeces, black colour, foul smelling, watery etc. The age of the animals was between 15 days and 12 years. Animals with 15 days to 2.5 years were considered as young. Sampled animals had different physical profiles (extremely weak, weak, normal and apparently healthy). In another set of sampling, 14 samples were collected from cowshed for stray cattle (Gaushala). These animals typically had shooting diarrhoea from 6 months to 2 to 3 years, were extremely weak, emaciated, cachectic and some even in lateral recumbence. Samples were also collected from 8 apparently healthy buffaloes from an organised dairy farm at Hisar, Haryana.

Plasma was separated and stored at −20 °C till further analysis. To assess the status of MAP infection, ELISA was performed using in-house i-ELISA on plasma samples, and acid-fast staining, culture and IS*900* PCR on faecal samples. IS*900* PCR was also performed on whole blood samples. PBMCs were harvested from EDTA blood samples. Trizol was added to the process for RNA extraction. Animals positive for i-ELISA were reported as seropositive. Animals positive in three tests were considered positive for MAP infection. Plasma samples from diarrhoeic animals were negative in the ELISA culture. Microscopy and PCR were taken as negative controls. 

### 2.2. Microscopic Examination (Acid Fast Staining)

The test was conducted as described previously [18].

### 2.3. ELISA Test

The test was performed using an indigenous ELISA kit (i-ELISA, Sigma-Aldrich, St. Louis, MO, USA) supplied by S.V. Singh, as per the method of Milner et al. (1987) [19], using plasma as a sample. Sample-to-positive ratios were derived to estimate the corresponding status of MAP infection as per Collins (2002) (strong-positive, positive, low-positive, suspected, and negative for MAP infection (Supplementary Table S1).

### 2.4. Faecal DNA Isolation

Faecal DNA isolation is done using the method described by van Embden et al. (1993) [20]. The faecal sample (2.0 g) was homogenised in sterilised PBS and centrifuged at 3500 rpm for 45 min at room temperature (RT). The supernatant was discarded; the middle semi-solid layer of the concentrated faecal samples was taken and inoculated into 300 µL of sterilised PBS. The resulting material was subjected to heating at 95 °C for 10 min. After this, 50 μL of lysozyme (20 mg/mL) was added and mixed, followed by incubation at 37 °C for 2 h. Then 50 μL of 10% SDS and 20 μL of proteinase K (10 mg/mL) were added, followed by incubation at 56 °C for 2 h. Then 65 μL of CTAB/NaCl (preheated to 65 °C) was added and incubated at 65 °C for 30 min. After incubation, an equal volume of Chloroform-isoamyl alcohol (24:1) was added and vortexed. The suspension was incubated at room temperature for 5 min and was then centrifuged at 10,000 rpm for 20 min. After centrifugation, the aqueous layer was transferred to a sterile Eppendorf tube. To the aqueous layer, 0.6 mL of isopropyl alcohol was added and kept at −20 °C for overnight. After precipitation, centrifugation was done at 10,000 rpm for 20 min. The supernatant was discarded, and sediment was washed twice with 1 mL of 70% ethanol by centrifugation at 10,000 rpm for 20 min. The pellet was allowed to air dry. The DNA pellet was re-suspended in 30 μL TE buffer, then kept at 4 °C for 1 h to allow for the dissolution of the pellet and then stored at −20 °C for further use.

### 2.5. DNA Isolation from Whole Blood

DNA isolation from blood and PCR was done as described previously [18].

### 2.6. IS900 PCR

IS*900* PCR was performed as per Marsh et al. (1999) [21] using P-90B and P-91B primers with some modifications [18]. ‘MAP strain’ positive control DNA was kindly provided by the Department of Biotechnology, GLA University, Dist. Mathura, Uttar Pradesh. The presence and yield of specific PCR product (413 bp) were analysed by 1.0% agarose gel electrophoresis with positive (MAP ‘S5’ Bison type) and negative (DNase free water) control.

### 2.7. Reverse Transcription and Real-Time PCR

PBMCs were harvested from whole blood using equal volumes of Histopaque 1077 (Sigma-Aldrich, Dorset, U.K.) (1:1), added to the blood and then centrifuged at 3500 rpm for 30 min. The buffy coat layer containing PBMCs was washed with IX PBS (pH-7.2) twice, counted using a hemocytometer (using trypan blue, live dead staining) and stored at −80 °C until further use. Total RNA was extracted from the isolated PBMCs using the Trizol Method. The quantity and quality (*A*_260/230_ and *A*_260/280_) of total RNA were determined using the spectrophotometer. cDNA (Complementary DNA) was synthesised as per the protocol described by the manufacturer (Fermentas, Hanover, NH, USA) using Oligo dT and subjected to qRT-PCR for expression analysis of immunoinhibitory receptors, TIM-3 and PD-1.

qRT-PCR was performed in a 20 μLreaction volume containing 4 μL (100 ng/μL)of diluted cDNA and 16 μL of master mix composed of 10 μL of 2X SYBR Green, 0.5 μL each of 10 μM forward and reverse primers, and 5 μL of nuclease-free water (Supplementary Table S2) [22,23]. Each qRT-PCR reaction was performed in duplicate to check the quality by assessing intra-assay variation. The amplification was carried out in a 96-well block using a Quanta studio qRT-PCR instrument (Thermo Fisher Scientific Inc, Waltham, MA, USA) with the following conditions: 45 cycles of 15 s at 95 °C (denaturation), 30 s at 55 °C (annealing) and 30 s at 72 °C (extension) (Supplementary Tables S3 and S4) [24]. In order to evaluate the quality of qRT-PCR reactions in terms of nonspecific amplification and primer-dimer formation, a dissociation curve for each gene was obtained by increasing the temperature from 60 °C to 95 °C. Melting temperature and CT values of primers are detailed in Supplementary Table S5 [25]. The relative fold change was calculated using CT values normalised with GAPDH via the ΔCT formula. Animals exhibiting a fold change above one showed relatively high expression of PD-1 and TIM-3 in comparison to the control. However, the normalised CT values for all samples are available in Supplementary Table S6.

### 2.8. Statistical Analysis

Statistical analysis was done using Prism Pad 9 software, GraphPad, Boston, MA, USA. One-way ANOVA, Tukey’s multiple comparison test and t-tests were performed.

## 3. Results

### 3.1. Expression of TIM-3 (T Cell Immunoglobulin Mucin Protein 3) on Buffalo PBMCs

**Figure 1:**
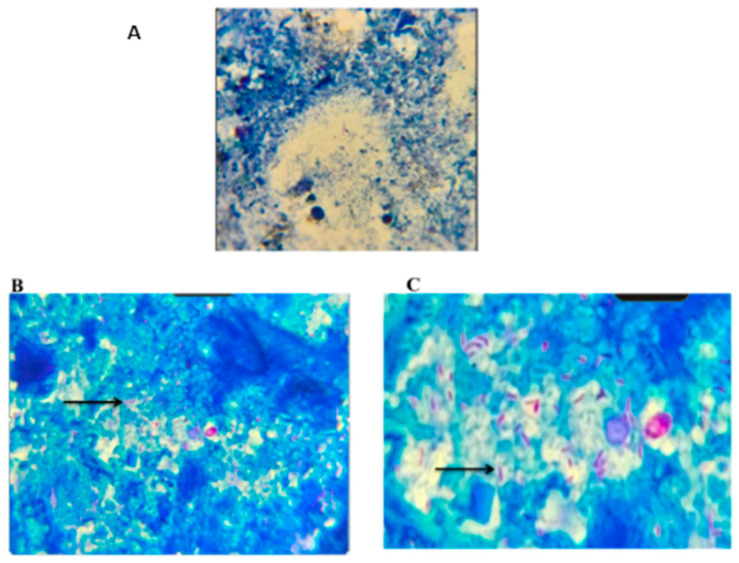
Microscopic examination of acid-fast bacilli (MAP) under oil immersion 100×. (**A**) Absence of MAP bacilli; (**B**,**C**) Arrow indicates the presence of MAP bacilli.

**Figure 2:**
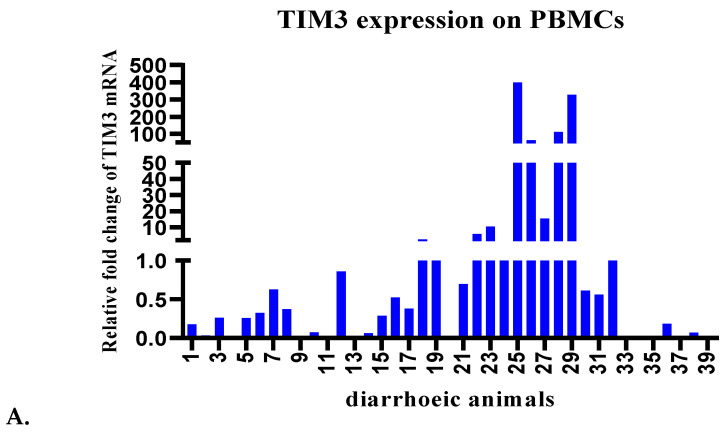

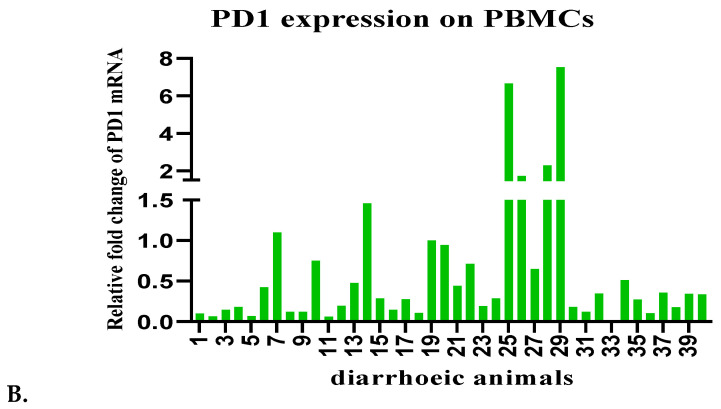
(**A**) TIM-3 expression on PBMCs of 40 animals by qRT-PCR. Animal numbers 17–24 were apparently healthy buffaloes from an organised dairy farm at Hisar, Haryana. Animal numbers 1–16 and 25–40 are the diarrhoeic bovines. Animals 25–29 were strongly positive in ELISA, microscopy and IS*900* faecal PCR. One seronegative (negative in microscopy and IS*900* PCR), apparently healthy buffalo from an organised dairy farm at Hisar, Haryana, was included as the control sample for evaluating fold change in TIM3 expression. (**B**) PD-1 expression on PBMCs of 40 animals by qRT-PCR. Animal numbers are the same as described above. One seronegative (negative in microscopy and IS*900* PCR), apparently healthy buffalo from an organised dairy farm at Hisar, Haryana, was included as the control sample for evaluating fold change in PD-1 expression.

Ex-vivo experiments were performed with PBMCs harvested from whole blood from a total of 40 animals (screened by ELISA and acid-fast staining, as shown in Figure 1) to determine whether TIM-3 is expressed on bovine PBMCs. Eight animals apparently healthy were from an organised dairy farm at Hisar, Haryana and 32 were suspected diarrhoeic bovines (suspected for MAP) reporting to the LUVAS veterinary clinical complex (Supplementary Table S5) for therapeutic intervention. Standardisations for evaluating the expression of TIM-3 on buffalo PBMCs were done using both the set of published primers as well as the set of self-designed primers (Supplementary Tables S4 and S5). qRT-PCR was performed and shown in Figure 2A (in animal numbers 25–29), the relative TIM-3 expression was found to be around 400-fold, 330-fold, 112-fold, 65-fold and 16-fold higher in 5 chronically diarrhoeic PBMC samples (MAP-seropositive), compared to healthy controls (animal numbers 17–24) and the expression was higher than other diarrhoeic animals that were MAP-seronegative.

### 3.2. Expression of PD-1 on Buffalo PBMCs

**Figure 3:**
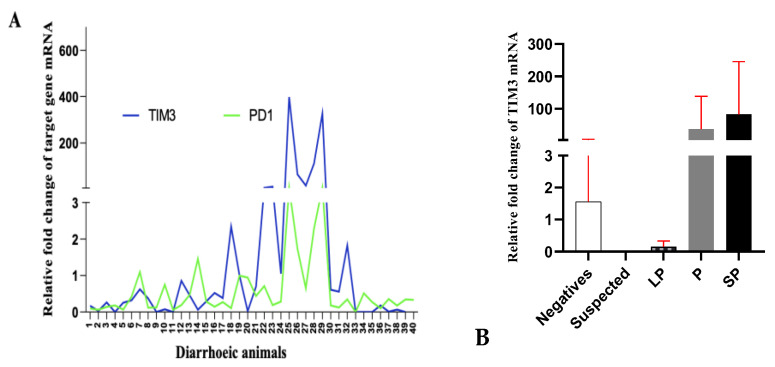
(**A**) TIM-3 and PD-1 are co-expressed on buffalo PBMCs: TIM-3 and PD-1 expression was evaluated by qRT-PCR on PBMCs of 40 animals. (**B**) Blood (with anticoagulant) was harvested from diarrhoeic bovine, and plasma was subjected to i-ELISA (as described in materials and method). Based on the S/P ratio obtained, animals were classified as MAP-seronegative, suspected, low positive, positive and strongly positive. Fold change in TIM-3 expression level compared to the TIM-3 expression on apparently healthy, MAP-seronegative (negative in microscopy and IS*900* PCR) was evaluated on the PBMC fraction of these animals by qRT-PCR.

TIM-3 is expressed by effector T cells, and PD-1 is expressed by various immune cells that include lymphocytes, myeloid cells and macrophages. PD-1 expression was evaluated on PBMCs harvested from the above-mentioned 40 animals. Standardisations for evaluating expression of PD-1 on buffalo PBMCs were done using both the set of published primers as well as the set of self-designed primers (Supplementary Tables S4 and S5). qRT-PCR was performed (as discussed in the materials and methods section), and we could detect PD-1 expression on the PBMCs from healthy as well as diarrhoeic animals (Figure 2B). As shown in Figure 2B, relatively higher expression levels of PD-1, i.e., around 7-fold, 1.75-fold, 2.5-fold, and 7.6-fold higher, were recorded in 4 chronically diarrhoeic animals (animal numbers 25, 26, 28 and 29) were MAP-seropositive, compared to healthy controls (animal numbers 17–24) and the expression was higher than other diarrhoeic animals that were MAP-seronegative animals that reported to clinics for therapeutic management of diarrhoea. Interestingly, relatively higher PD-1 levels were recorded in those 4 animals that had also exhibited relatively high levels of TIM-3 expression, suggesting a state of high TIM-3 and PD-1 co-expression on the PBMC fraction of these animals. TIM-3 is known to be transiently expressed by T cells after acute infection, whereas high TIM-3 expression is retained throughout chronic infection and high TIM-3 and PD-1 co-expression has been associated with a more severe exhaustion. Thus suggesting a state of exhaustion in chronically diarrhoeic MAP-seropositive animals. Evaluation of the levels of such immuno-inhibitory receptors in chronic infections may help in identifying an ongoing state of immune exhaustion. Furthermore, the levels of PD-1 expression in four high-expressing diarrhoeic buffaloes (animal numbers 25, 26, 28 and 29) were lower compared to the levels of TIM-3 expression in these animals (Figure 3A).

### 3.3. High Co-Expression of TIM-3 Correlates with MAP-Seropositivity

Our data indicates that both TIM-3 and PD-1 are detectable on buffalo PBMCs. Furthermore, the expression levels of PD-1 did not coincide with the levels of TIM-3; however, in chronically diarrhoeic animals with high TIM-3 expression, PD-1 expression levels were also high and coincided with high TIM-3 expression (Figure 3A), suggesting high co-expression in chronically diarrhoeic MAP-seropositive animals. High co-expression of TIM-3 and PD-1 correlates with more severe exhaustion, as these animals presented chronic diarrhoea for several months in the clinics, with features of emaciation. To evaluate whether TIM-3 levels correlated with MAP-serological status, based on the S/P ratio (as described in the methods section), the animals were categorised as MAP-negative, MAP-suspected, MAP-low-positive, MAP-positive and MAP-strongly-positive. High TIM-3 expression was observed on MAP-positive and MAP-strong-positive animals compared to MAP-low-positive and MAP-negative animals; however, the difference in TIM-3 expression levels could not reach the level of significance due to a comparatively smaller number of animals in either of the categories and due to large individual variations (Figure 3B).

### 3.4. High Co-Expression of TIM-3 and PD-1 in Chronically Diarrheic, Emaciated and Debilitated Cattle

**Figure 4:**
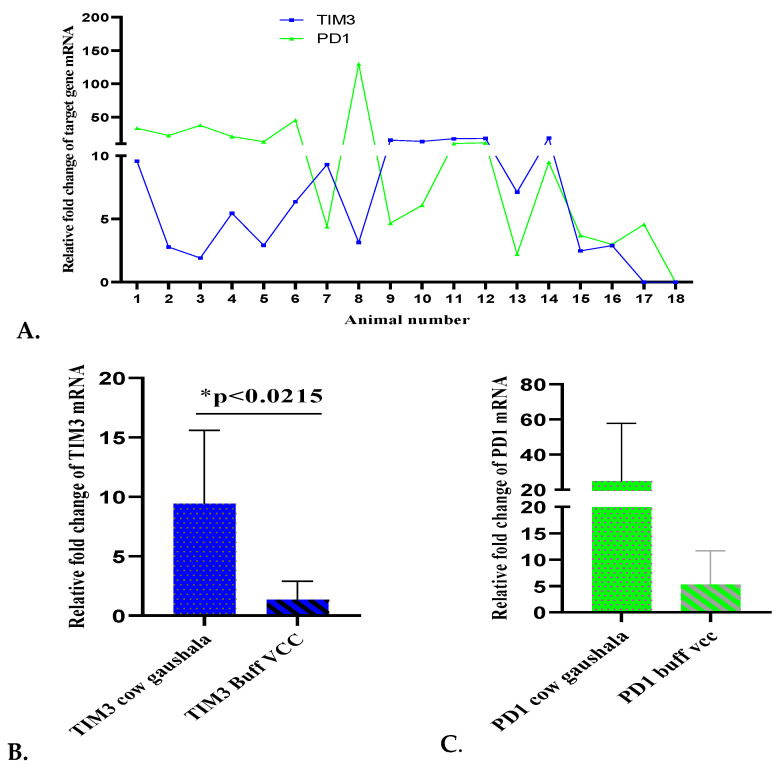
High co-expression of TIM3 and PD-1 on stray cattle PBMCs: TIM-3 and PD-1 expression was evaluated by qRT-PCR on PBMCs of stray cattle. (**A**) High TIM-3 and PD-1 co-expression on stray cattle PBMCs (1–14) (MAP-seropositive), 15–17 are chronic diarrhoeic buffaloes (MAP-seronegative), 18-acutely diarrhoeic buffalo. Comparison of TIM-3 (**B**) and PD-1 (**C**) expression on diarrhoeic buffaloes at VCC (Veterinary Clinical Complex of Lala Lajpat Rai University of Veterinary and Animal Sciences, Hisar, Haryana, India) and stray cattle.Pairwise statistical comparison were performed using Student’s *t*-test (* = *p* < 0.05).

**Figure 5:**
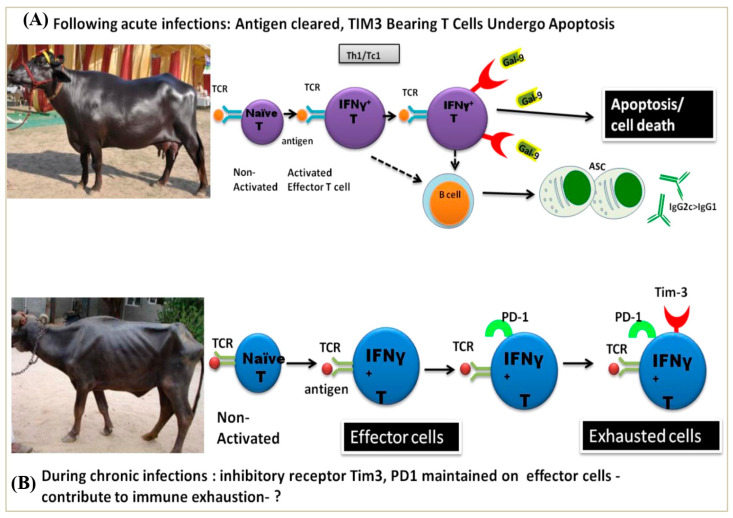
(**A**) Upon activation, naive cells become activated effector T cells. These cells provide help to B cells. Activated effector T cells upregulate TIM-3 (T cell immunoglobulin mucin protein-3) (an immuno-inhibitory receptor), which, upon binding to its ligand galectin 9 (expressed on innate cells and other immune cells), results in apoptosis of TIM-3 bearing effector T cells. This results in dampening of the immune response, which might otherwise result in immunopathology due to excessive cytokine production by the immune effector cells. The remaining cells down-regulate TIM-3 after the peak of an immune response. (**B**) However, in certain situations (chronic conditions), TIM-3 and PD-1 (programmed death receptor-1) are persistently highly expressed in T cells and such cells are identified as exhausted cells, as we have reported in our current study.

We next decided to evaluate immuno-inhibitory receptors (TIM-3 and PD-1) expression on the PBMCs from chronically diarrheic, emaciated, cachectic, debilitated and almost laterally recumbent cattle located at cow shed for stray cattle, commonly known as “gaushala” to confirm whether MAP-seropositive cows also Co-express TIM-3 and PD-1. Whole blood was obtained from 14 cattle (#1–14 in Figure 4A), and reverse transcription and qRT-PCR were performed on the PBMC fraction to obtain the expression levels of TIM-3 and PD-1 (Figure 4A). At the same time, three chronically diarrhoeic and one acutely diarrhoeic buffalo were also reported to VCC, LUVAS, for therapeutic intervention. We therefore compared TIM-3 and PD-1 expression on cattle PBMC (from gaushala) to TIM-3 and PD-1 expression on Buffalo PBMC (VCC, LUVAS). As shown in Figure 4A, gaushala cattle PBMCs expressed high levels of TIM-3 and PD-1 (as shown as fold change compared to the expression of PBMCs from apparently healthy MAP-seronegative animals from an organised dairy farm at Hisar, Haryana). Further, the three chronically diarrhoeic MAP-seropositive buffaloes (#15, 16, 17 in Figure 4A) expressed higher TIM-3 and/PD-1 compared to healthy, but the levels were lower than those expressed on gaushala cattle PBMCs (Figure 4B,C). TIM-3 and PD-1 on one acutely diarrheic MAP-seronegative buffalo PBMC (# 18 in Figure 4A) were barely detectable, suggesting their strong expression on chronically diarrheic MAP-seropositive emaciated, cachectic and debilitated and almost laterally recumbent cattle (Figure 5). Our data suggests high TIM-3 and PD-1 (negative regulators of T cell responses co-expression in the peripheral blood of both MAP-seropositive buffaloes and cattle. Blocking both TIM-3 and PD-1 could restore immune responses in these animals.

## 4. Discussion

Diarrheic animals are treated symptomatically with anti-diarrheal drugs; however, in several cases, animals do not respond to therapy and slowly progress to emaciation. Certain pathogens can persist for a long time by inducing immune exhaustion. We aimed to evaluate immuno-inhibitory receptors (exhaustion markers) on the PBMCs of diarrheic bovines (mainly in diarrheic buffaloes reporting to VCC for therapy and suspected to be suffering from MAP infection). Our results suggested that relative TIM3 expression was 400, 330, 112, 65 and 16-fold higher in the PBMCs samples of 5 buffaloes, chronically diarrheic and seropositive for MAP infection, as compared to healthy controls and other diarrheic animals that were seronegative. Comparatively higher expression of PD-1 was around 7, 1.75, 2.5, and 7.6-fold higher in 4 chronically diarrheic animals (animal numbers 25, 26, 28 and 29) that were seropositive for MAP infection, as compared to healthy controls and other MAP-seronegative diarrhoeic animals that reported to clinics for therapeutic management of diarrhoea. High expression of TIM-3 correlated with MAP-seropositivity. High co-expression of TIM-3 and PD-1 was also observed in chronically diarrheic, emaciated, debilitated and recumbent MAP-seropositive cows (representing advanced clinical stage) housed in stray cattle sheds (gaushala).

Our data indicated that TIM-3 and PD-1 are expressed in apparently healthy as well as diarrhoeic buffaloes. However, TIM-3 and PD-1 cooperation was strongly increased on PBMCs from MAP-seropositive chronically diarrhoeic buffaloes. Antigen persistence and constant antigenic stimulation, along with increased expression of immuno-inhibitory receptors (PD-1, LAG-3, and TIM-3) and chronic TCR activation, have been reported in a state of exhaustion [26]. In cattle, T cell exhaustion has been observed in chronic infections, such as MAP [17], where selective up-regulation of TIM-3 is demonstrated on the exhausted T cells [27,28]. Furthermore, the cooperation of TIM-3 and PD-1 (Programmed cell death protein-1) results in more severe exhaustion [29]. However, immune exhaustion and the molecular mechanisms of this suppressive state in livestock animals and, in particular, buffaloes are poorly understood and have not been reported in chronic infections of buffaloes, especially MAP.

In our previous study, we reported a high incidence of *paratuberculosis* infection in diarrhoeic animals [18], with decreased leukocytic counts [30] in MAP-seropositive and MAP-PCR-positive buffaloes, suggesting the chronic nature of the disease. Our current study suggesting increased expression of immuno-inhibitory receptors, along with our previous finding of diminished leukocytic population in MAP-positive animals, could explain the phenomenon of immune exhaustion and pathogen persistence and the chronic nature of *paratuberculosis* in buffaloes. Interestingly, strong positive buffaloes in i-ELISA expressed the highest levels of TIM-3 compared to low positive, suspected or negative animals. However, the difference was not significant in various groups. This may be due to the lesser number of buffaloes in some sero-groups and huge variations in individual buffaloes. Incidence of immune exhaustion could be depicted by various experimental techniques such as flow cytometry, intracellular cytokine assay (ICCS), Detection of recall responses, IFN-γ release assay (IGRA), including evaluation of expression levels of immuno-inhibitory receptors.

Stray cattle from Gaushala were also included in our study. MAP-seropositive, chronically diarrhoeic, extremely weak, recumbent and cachectic cows (representing an advanced clinical stage) also exhibited a high co-expression of TIM-3 and PD-1 on their PBMC fraction. In a single experiment, when the fold change (compared to apparently healthy and MAP-seronegative and PCR-negative bovine) in TIM-3 and PD-1 were compared in stray chronically diarrhoeic, extremely weak and cachectic cows with diarrhoeic buffaloes reporting at VCC, stray cattle exhibited greater immunoinhibitory receptor expression compared to diarrhoeic buffaloes at VCC. Furthermore, in stray cattle, PD1 expression was higher than TIM-3 in at least 50 per cent of animals, whereas the trend was somewhat opposite in the case of diarrhoeic buffaloes at VCC (with TIM-3 expression exceeding PD1 expression). It was clear from the clinical presentation that stray cattle (MAP-seropositive) were in a more advanced clinical stage of *paratuberculosis* compared to diarrhoeic buffaloes brought to VCC. Thus, our study could mean that in progress towards exhaustive phenotype in *paratuberculosis*, comparatively in the initial stages, TIM-3 expression exceeds PD-1 expression; however, as the disease progresses, PD-1 expression overtakes TIM-3 expression. Future longitudinal studies are required to further evaluate relative TIM-3/PD-1 expression in MAP-seropositive bovines, and, if the hypothesis is confirmed by experimental evidence, could serve as a marker to evaluate the stage of *paratuberculosis*.

We used i-ELISA to assign MAP-serological status in our study. ELISA is the most widely used immunological test to measure anti-MAP antibodies in serum and milk samples. This test is highly desirable as a ‘Screening Test’ due to the ease of sampling of input samples (blood or milk), rapidity of the test (few hours), and the large number of samples that could be screened at the same time and repeatability at short intervals and low cost of the test. However, reported sensitivity for detecting sub-clinically infected animals may be lower than the ‘faecal culture’. Most of the studies in India and internationally reported variable sensitivity of serum ELISA ranging from 15 to 75%. The likelihood ratio method has been used for the standardisation of ‘i-ELISA’ as a kit (utilised in our experiments) at CIRG Makhdoom [31,32]. Data from bacterial culture may be required when apparently healthy animals or animals from low-prevalence herds test positive for ELISA. Re-examination of false negative animals should be carried out in 6–12 months. ELISA is a preferred diagnostic and screening test due to its advantages, such as easy automation, repeatability and the possibility of evaluating a large number of samples together. ELISA is comparatively inexpensive and has very good sensitivity and specificity in clinical stages. ELISA may have disadvantages in cases where the antigen used is not from a native source and has been procured from a different source, and age differences in animals tested may lead to sensitivity and specificity issues. 

There were certain limitations in our study, especially the low sample number of buffaloes. A higher number of MAP-seropositive animals is required in the study to ascertain the certainty of our data. Further, analysis in the context of age, parity, and stage of lactation are other factors that need attention. The current study did not include a longitudinal study on the same animals to evaluate immuno-inhibitory receptor expression in the same animal over time. Such a type of study might involve regular sampling from large ruminants, which is cumbersome due to the non-availability of animals, owner’s reluctance, etc. *Bos taurus* and *Bubalus bubalis* are two different species included in the family Bovidae and seem quite similar. However, physiological, behavioural and anatomical differences exist among *Bos taurus*, *Bos indicus*, and the buffalo [33], and thus, comparative evaluation of immuno-inhibitory receptors in these MAP-seropositive and negative cattle and buffaloes might provide even more elaborated insights. Furthermore, our study was conducted on naturally infected bovines in a clinical setting; thus, it is highly likely that co-infections may exist along with MAP. However, it is very difficult to ascertain the history of infection in animals and rule out each and every infection/non-infectious cause of diarrhoea.

Nonetheless, our study provided a clue on the correlation of immuno-inhibitory receptor expression with MAP-serological status and disease severity. While the study provides valuable insights into the correlation between immuno-inhibitory receptor expression and MAP-serological status, it cannot definitively determine whether TIM-3 and PD-1 expression are related to the cause or consequence of chronic MAP manifestations. Our study also provides a stage for planning and development of strategies to reverse exhaustion and promote antigen clearance. The major question is whether we could reverse exhaustion in buffaloes and how to improve livestock health. Blockade of immuno-inhibitory interaction, manipulating regulatory T cell activity, cytokine therapy or administration of herbal preparations that boost immune responses are some of the strategies that must be experimentally evaluated in large ruminants to reverse the exhaustive state and improve animal productivity and ultimately increase farmer’s income.

Animals suffering from chronic diarrhoea are treated with classical anti-diarrhoeal therapy, but the animals do not respond to therapy, and the diarrhoeic state continues, resulting in progressive weakness leading to cachexia. It is thus crucial to delineate whether exhaustion occurs and novel therapeutic strategies, for example, discovering a host-directed antidiarrhoeal that targets the hosts’ immuno-inhibitory interaction (that targets checkpoint blockade of immuno-inhibitory interactions) for augmentation of immunity and reverse the state of exhaustion, can lead to the paradigm shift in the management of *paratuberculosis*. To achieve this, emphasis on the small molecule inhibitors or other such molecules that could effectively regulate immunity along with minimal side effects might be useful. Answering such questions may transform therapeutic modalities for chronic and persistent infections in ruminants [34]. The literature available on ruminant immunology is very scanty, and the majority of studies are done in experimental infection models, which does not translate well in the natural clinical setting where huge individual variations in immune responses exist from animal to animal, possibly due to heterologous immunity [35].

## 5. Conclusions

To the best of our knowledge, this is the first study evaluating the expression of immuno-inhibitory receptors on the PBMC fraction of suspected, spontaneous, naturally infected MAP-seropositive diarrheic buffaloes and stray cattle. Our data indicate that TIM-3 and PD-1 were expressed in apparently healthy as well as diarrhoeic buffaloes. However, the co-expression of TIM-3 and PD-1 has strongly increased in PBMCs from MAP-seropositive chronically diarrhoeic buffaloes. MAP-seropositive, chronically diarrhoeic, extremely weak, recumbent and cachectic cows (representing an advanced clinical stage) also exhibited a high co-expression of TIM-3 and PD-1 on their PBMC fractions. Samples from diarrheic buffaloes at VCC represented high TIM-3 compared to PD-1; however, in stray cattle, at least 50% of animals had high PD-1 compared to TIM-3. Study suggested the possible role of exhausted immune cells in promoting the chronic progression of Johne’s disease in the natural clinical setting where huge individual variations in immune responses exist from animal to animal, possibly due to heterologous immunity and individual infection history of the animal.

## Data Availability

All the data are available within this manuscript and in Supplementary Materials file.

## Supplementary Materials

The following supporting information can be downloaded at:https://www.mdpi.com/article/10.3390/pathogens13060473/s1, Table S1: S/P ratios and corresponding status of buffaloes and cattle with respect to JD using ‘i_ELISA’; Table S2: PCR Reaction Mixture for TIM-3 and PD-1; Table S3: PCR Reaction: cycle conditions; Table S4: Evaluation and standardization of TIM-3 primers: A set of TIM-3 primers were designed and another set of published primers were evaluated to record the expression of TIM3 on bovine PBMCs; Table S5: PBMCs were harvested from apparently healthy animals or diarrhoeic bovines for standardizations of TIM-3 expression on PBMC fraction by real time PCR assay. PBMCs harvested from a cat (to confirm amplification by bovine specific primers) reporting at VCC or NFW (nuclease free water) were used as negative controls. Both healthy and diarrhoeic bovines expressed TIM-3; Table S6: Age, Species and characteristics of animals selected for study.
