# Supplementary material for: Evaluation of Immune Exhaustion and Co-Inhibitory Receptor Expression in Mycobacterium avium Subspecies paratuberculosis (MAP) Seropositive Diarrhoeic Bovines
Source: Pathogens. 2024 Jun 4;13(6):473. doi: 10.3390/pathogens13060473 (PMC11206971; doi:10.3390/pathogens13060473)
Supplement: Supplementary file 1 [file pathogens-13-00473-s001.zip › pathogens-2961400-supplementary.pdf]

Supplementary Table 1: S/P ratios and corresponding status of buffaloes and cattle with respect to JD using 'i-ELISA'

S. No.	Calculated value of S/P Ratio	Status of Johne's disease in animals	Status
1	0.00 – 0.09	Negative (N)	Negative
2	0.10 – 0.24	Suspected or Borderline (S)	
3	0.25 – 0.39	Low Positive (LP)	
4	0.4 – 0.99	Positive (P)	Positive
5	1.0 – 10.0	Strong Positive (SP)	

MAP infection S/P ratio categories: N, Negatives; S, Suspected; LP, Low positive; P, Positive; and SP, Strong positive for MAP infection; N+S+LP, Negative (Healthy); and P+SP, Positive (Infected).

Supplementary Table 2: PCR Reaction Mixture for TIM-3 and PD-1

Reagents	Vol (µl)
2x SYBR Green	10
Forward primer (10µM)	0.5
Reverse primer (10µM)	0.5
Nuclease Free Water	5
DNA template	4
**Original reaction volume**	**20 µl**

Supplementary Table 3: PCR Reaction: cycle conditions

Steps	Process	Temperature (°C)	Duration	Cycle
1.	Initial Denaturation	95	10 min	1 cycle
2.	Denaturation	95	15 sec	
3.	Annealing	55	30 sec	45 cycles
4.	Extension	72	30 sec	
5.	Final Extension	72	10 min	1 cycle

Supplementary Table 4: Evaluation and standardization of TIM3 primers: A set of TIM3 primers were designed and another set of published primers were evaluated to record the expression of TIM3 on bovine PBMCs.

Target gene	Primer name	Sequence (5'-3')	References
**Bovine TIM3**	TIM3-PP1-FP	GGA TCC AAT TCC CAG GTC TAA	(3)
	TIM3-PP1-RP	AGG GTC TTC AGT GTC CGT GT	
	TIM3-PP2-FP	GGA TGC TCA CCA CAA AGG GA	NA
	TIM3-PP2-RP	AAG AAT CAG AGC CAG CCC AG	
**Bovine PD1**	PD1-PP1-FP	AAT GAC AGC GGC GTC TAC TT	(4)
	PD1-PP1-RP	GAT GAC CAG GCT CTG CAT CT	
	PD1-PP2-FP	CTG TCA CAG TGG ACT ACG GG	NA
	PD1-PP2-RP	TGT CCA TCC TCG GTC CTC AG	
**Bovine β-actin**	β-actin-FP	CCC CAG CCA TGT ACG TTG CTA TCC	(2)
	β-actin-RP	GCC TCA GGG CAG CGG AAC CGC TCA	

1. **Singh, P.K., Singh S.V., Kumar H., Sohal J.S., Singh, A.V.,** 2010. Diagnostic Application of IS900 PCR Using Blood as a Source Sample for the Detection of Mycobacterium avium Subspecies Paratuberculosis in Early and Subclinical Cases of Caprine Paratuberculosis. Veterinary medicine international **2010:**748621.
2. **Kumar, R., Chander, Y., Khandelwal, N., Verma, A., Rawat, K.D., Shringi, B.N., Pal, Y., Tripathi, B.N., Barua, S., Kumar, N.,** 2022. ROCK1/MLC2 inhibition induces decay of viral mRNA in BPXV infected cells. Sci Rep **12:**17811.
3. **Okagawa, T., Konnai, S., Ikebuchi, R., Suzuki, S., Shirai, T., Sunden, Y., Onuma, M., Murata, S., Ohashi, K.,** 2012. Increased bovine Tim-3 and its ligand expressions during bovine leukemia virus infection. Veterinary Research **43:**45.
4. **Ikebuchi, R., Konnai, S., Sunden, Y., Onuma, M., Ohashi, K.,** 2010. Molecular cloning and expression analysis of bovine programmed death-1. Microbiology and immunology **54:**291-298.

Supplementary Table 5: PBMCs were harvested from apparently healthy animals or diarrhoeic bovines for standardizations of TIM3 expression on PBMC fraction by real time PCR assay. PBMCs harvested from a cat (to confirm amplification by bovine specific primers) reporting at VCC or NFW (nuclease free water) were used as negative controls. Both healthy and diarrhoeic bovines expressed TIM3.

Target Name	Sample Name	Ct	Tm1
PD1-1	Bovine -PBMC-1	37.066	87.934
	Bovine -PBMC-1	35.478	88.096
	Negative Control	Undetermined	72.344
PD1-2	Bovine -PBMC-1	30.447	88.746
	Bovine -PBMC-1	29.948	88.908
	Negative Control	Undetermined	60.002
TIM3-1	Bovine -PBMC-1	35.016	82.250
	Bovine -PBMC-1	34.589	82.250
	Negative Control	Undetermined	72.019
TIM3-2	Bovine -PBMC-1	30.722	82.250
	Bovine -PBMC-1	31.607	81.763
	Negative Control	Undetermined	60.002

Supplementary Table 6: Animal details

(CT value and relative fold change of target gene of all animals)

S.No	Species	CT		TIM3	PD1
		TIM3	PD1		
1	Buffalo, 4yrs	11.1	10.8	0.174	0.09971683
2	Buffalo	13.5	11.4	0.033	0.06411892
3	Buffalo,9 yrs	10.5	10.2	0.264	0.14454962
4	Buffalo, 4yrs	17.4	9.8	0.002	0.18343065
5	Buffalo, 3yrs	10.6	11.3	0.256	0.06903596
6	Buffalo, 5yrs	10.2	8.7	0.324	0.42424429
7	Cow, 6yrs	9.3	7.3	0.624	1.09747620

8	Buffalo, 3.5 months	10.0	10.4	0.375	0.12258313
9	Buffalo	22.0	10.4	0.000	0.12357947
10	Cow, 7yrs	12.3	7.8	0.076	0.75215000
11	Buffalo, 4yrs	17.9	11.5	0.002	0.05975987
12	Buffalo, 3yrs	8.8	9.8	0.859	0.19259982
13	Buffalo, 5 months	ND	8.5	ND	0.47331412
14	Buffalo, 7yrs	12.6	6.9	0.062	1.45760725
15	Buffalo, 6 months	10.4	9.2	0.291	0.28589585
16	Buffalo, 4yrs	9.5	10.2	0.526	0.14682060
17	Buffalo, 4yrs	10.0	9.3	0.382	0.27389790
18	Buffalo, 12 yrs	7.4	10.7	2.346	0.10739710
19	Buffalo,11yrs	8.6	7.4	1.000	0.99990485
20	Buffalo, 7 yrs	14.1	7.5	0.023	0.94451805
21	Buffalo, 7yrs	9.2	8.6	0.699	0.43986484
22	Buffalo, 7yrs	6.1	7.9	5.735	0.71102528
23	Buffalo, 7yrs	5.2	9.8	10.560	0.19189134
24	Buffalo, 6yrs	8.5	9.2	1.052	0.28601305
25	Buffalo, 7yrs	0.0	4.7	398.357	6.67144835
26	Buffalo, 4yrs	2.6	6.6	63.881	1.73147313
27	Buffalo, 6yrs	4.6	8.1	15.519	0.65022007
28	Cow, 8yrs	1.8	6.2	111.356	2.29262437
29	Buffalo, 4yrs	0.2	4.5	327.120	7.54227629
30	Buffalo	9.3	9.9	0.613	0.18237754
31	Buffalo	9.4	10.4	0.560	0.12336457

32	Buffalo	7.7	8.9	1.828	0.34844431
33	Buffalo	22.6	16.5	ND*	0.00187782
34	Buffalo	19.1	8.4	0.001	0.51350163
35	Buffalo	17.7	9.3	0.002	0.27246335
36	Buffalo, 4yrs	11.1	10.7	0.183	0.10313628
37	Buffalo, 3yrs	15.1	8.9	0.011	0.35917065
38	Buffalo	12.4	9.9	0.070	0.17795878
39	Buffalo	23.2	8.9	0.000	0.34226283
40	Buffalo, 10 yrs	ND	9.0	ND*	0.33495099

***ND: Not Detected**
